# Sex enhances adaptation by unlinking beneficial from detrimental mutations in experimental yeast populations

**DOI:** 10.1186/1471-2148-12-43

**Published:** 2012-03-30

**Authors:** Jeremy C Gray, Matthew R Goddard

**Affiliations:** 1 School of Biological Sciences, University of Auckland, Private Bag 92019, Auckland Mail Centre, Auckland 1142, New Zealand

## Abstract

**Background:**

The maintenance of sexuality is a classic problem in evolutionary biology because it is a less efficient mode of reproduction compared with asexuality; however, many organisms are sexual. Theoretical work suggests sex facilitates natural selection, and experimental data support this. However, there are fewer experimental studies that have attempted to determine the mechanisms underlying the advantage of sex. Two main classes of hypotheses have been proposed to explain its advantage: detrimental mutation clearance and beneficial mutation accumulation. Here we attempt to experimentally differentiate between these two classes by evolving *Saccharomyces cerevisiae *populations that differ only in their ability to undergo sex, and also manipulate mutation rate. We cannot manipulate the types of mutation that occur, but instead propagate populations in both stressful and permissive environments and assume that the extent of detrimental mutation clearance and beneficial mutation incorporation differs between them.

**Results:**

After 300 mitotic generations interspersed with 11 rounds of sex we found there was no change or difference in fitness between sexuals and asexuals propagated in the permissive environment, regardless of mutation rate. Sex conferred a greater extent of adaptation in the stressful environment, and wild-type and elevated mutation rate sexual populations adapted equivalently. However, the asexual populations with an elevated mutation rate appeared more retarded in their extent of adaptation compared to asexual wild-type populations.

**Conclusions:**

Sex provided no advantage in the permissive environment where beneficial mutations were rare. We could not evaluate if sex functioned to clear detrimental mutations more effectively or not here as no additional fitness load was observed in the mutator populations. However, in the stressful environment, where detrimental mutations were likely of more consequence, and where beneficial mutations were apparent, sex provided an advantage. In the stressful environment asexuals were increasingly constrained in their extent of adaptation with increasing mutation rate. Sex appeared to facilitate adaptation not just by more rapidly combining beneficial mutations, but also by unlinking beneficial from detrimental mutations: sex allowed selection to operate on both types of mutations more effectively compared to asexual populations.

## Background

The existence of sexual reproduction poses a conundrum because it is more costly than asexual reproduction, and over 100 years of thought has been directed toward understanding why sex is maintained [1-7]. The general idea that sex increases the efficacy of natural selection (the Weismann hypothesis) [1,4,8] is supported by an increasing amount of experimental data (reviewed in [9,10]). However, there are fewer experimental studies that allow us to decipher the mechanisms underlying the advantage of sex.

One long-standing leading theoretical explanation, based on Fisher and Muller's ideas (F-M), suggests that sex allows multiple beneficial mutations to simultaneously permeate populations, resulting in populations achieving combinations of beneficial mutations more rapidly than asexual populations. In contrast, asexual populations must rely on the slower stepwise fixation of individual mutations, which may hinder one another's spread through clonal interference when in different genomes [11,12]. Studies with a diversity of model systems have shown sexual populations to increase in fitness more rapidly than asexual ones, and the general inference is that this is a result of the F-M effect [9,13-20]. Some studies have provided a little more understanding of the mechanisms behind sex's advantage. Poon and Chao [21] demonstrated that recombination has a greater effect on fitness when genetic drift is larger, presumably since here negative linkage disequilibrium between beneficial mutations is greater, and thus sex serves to more effectively concentrate adaptive alleles. Work with *Chlamydomonas *[22] also supports the F-M hypothesis as it showed that sex had a greater effect on fitness gain in larger than smaller populations: in larger populations there are more likely to be multiple beneficial alleles segregating and thus sex may more effectively bring these together. Studies directly demonstrating that recombination speeds the fixation of beneficial mutations are rarer, but this has been shown in *Drosophila *[23] and bacterial models [24]. In sum, a number of studies, with a diversity of systems, suggest that sex and recombination serve to increase the rate of adaptation under a variety of situations, and this is presumed to be by more effectively bringing together beneficial mutations.

Whilst attractive, the downfall of ideas oriented around beneficial mutations is that any advantage to sex vanishes in the absence of directional selection. There is evidence to suggest that environmental stasis, where purifying rather than directional selection is important, may be the more common state in nature [25]. All populations are subject to detrimental mutations, whether they are adapting or not, and thus detrimental mutation clearance theories are potentially universally applicable. A stochastic based theory concerning the effect of sex on mutation clearance in small populations was originally proposed by Muller [26], and this idea has some experimental support [27]. The more general mutational deterministic (MD) hypothesis predicts that sex may be maintained since it serves to more effectively purge detrimental mutations in populations of any size [28]. Two conditions are needed for the MD hypothesis to counter the two-fold cost it theoretically imposes: 1, that the per-genome per-generation detrimental mutation rate (*U*) is above one; and 2, that detrimental mutations interact with negative epistasis [28]. These conditions seem rarely met in nature. Most, but not all, organisms surveyed have detrimental mutation rates below one [29]. The evidence for how detrimental mutations interact is less clear. Work with *Chlamydomonas *[30] and insects [31] suggest negative epistasis, but work with *E. coli *[32] and *Drosophila *[23] suggest approximately equal frequencies of positive and negative epistatic interactions. Experiments assessing the effect of sex on mutation clearance are fewer. A study by Zeyl and Bell [33] with *Saccharomyces cerevisiae *populations suggested that sex served to more effectively clear detrimental mutations than accumulate beneficial ones. In contrast, work with sexual and asexual yeast populations showed no difference in fitness under purifying selection [15], and Renaut *et al. *[34] examined the fate of sexual and asexual *Chlamydomonas *populations propagated under purifying selection and also found no evidence that sex more effectively cleared detrimental mutations. However, microbial detrimental mutation rates are very low (*U *≤ 0.001) [29,35,36] and so if the MD idea applies one might not have expected to see a measurable difference in equilibrium fitness between sexuals and asexuals in these experiments.

On balance it seems that beneficial mutation assemblage, rather than detrimental mutation removal, is the stronger evolutionary mechanism underlying the advantage of sex. However, since this is still not clear, it is of interest to attempt specific tests to disentangle the contributions of these processes. Theoretically, the benefit of sexual reproduction may be due to the simultaneous actions of both the F-M and MD mechanisms, but there have been few experimental tests for this. In asexual populations, any detrimental mutations linked to beneficial mutations can be expected to increase in frequency by "hitchhiking" along with them, so long as net genotype fitness positive [37]. This is predicted to increase the rate of molecular evolution, but lower the rate of adaptive evolution as a result of the decrease in effective population size due to the Hill-Robertson effect [38]. A similar argument has also been made that in asexual populations beneficial mutation spread may be suppressed since they likely reside in genomes that become increasingly full of "rubbish"; however, sex may liberate these beneficial mutations [39]. These ideas predict that asexual populations will show a tapering off in fitness gain under directional selection compared with sexual populations as beneficial mutations are not as effectively unlinked from increasingly detrimental backgrounds. This effect will be amplified under greater mutation pressure. Recently Morran *et al. *[19] showed that outcrossed nematode populations with increased mutation rates were able to adapt to a novel environment more rapidly than inbred populations, and they inferred that outcrossing both speeds adaptation and impedes the fixation of detrimental mutations compared with inbreeding. One study with *S. cerevisiae *manipulated mutation rates and compared the effect of sex in static and fluctuating environments [40], but we are unaware of experiments that have manipulated mutation pressure and compared sexual and asexual populations evolving under differing strengths of purifying and directional selection. Currently an experimenter may manipulate sexual status, mutation rate and environment of selection. However, the ideal experiment would also manipulate the types of mutations present in populations, and compare the effects of sex on the clearance of detrimental mutations and incorporation of beneficial mutations. We are far from having a large enough list of alleles with known differing fitness effects, even for the best characterized of model organisms. Even if there were a comprehensive list, the construction of starting populations with defined suites of characterized alleles, let alone tracking their change, is technically daunting.

Eukaryotic microbes present good model systems with which to attempt to test such questions as their mode of reproduction, mutation rates, and environment of growth may be easily manipulated. Yeast divides mitotically when supplied with sufficient nutrients, but starvation induces meiosis (sporulation) in diploids resulting in four haploid recombined spores. Each spore may be one of two mating types (a or α) as defined at a single Mendelian locus, and spores of the opposite mating type may mate once germinated. As meiosis in yeast and other microbial model systems is normally manipulated by starvation, this means that asexual and sexual replicates will experience different selection regimes. Moreover, starvation in both *Chlamydomonas *and *S. cerevisiae *is known to increase mutation rates [41,42], which will tend to increase genetic variation. Thus, these manipulations do not just have effects on the mode of reproduction but also on aspects that may alter the course of evolution independent of sexual reproduction. We employ a *S. cerevisiae *system that circumvents this issue as sexual and asexual populations experience identical conditions, including starvation, and differ only in their ability to engage in recombination, random assortment and syngamy [10,15]. Two genes required for normal recombination and meiosis were deleted to create the asexual sporulating strain used here. *SPO11 *encodes an endonuclease that initiates cross-over events by making double strand breaks in chromosomes: in its absence meiotic recombination does not occur [43]. *SPO13 *determines whether a cell goes through one or two meiotic divisions by altering the sister chromatid cohesion process [44]; in its absence only the second non-reductive meiotic division is achieved, and this results in the production of two diploid, as opposed to four haploid, spores. Because chiasmata are required to stabilize chromosome segregation, non-functional mutations of *SPO11 *would normally lead to aberrant chromosomal segregation, but this phenotype is rescued if *SPO13 *is non-functional as well, and leaves the asexual diploid double mutant fully fertile, producing diploid spores that are genetically identical to the parent cell [15,45]. The mitotic fitness effect of deleting these genes appears insignificant, and sporulation rates of sexual and asexual strains are equivalent [15].

Previous experiments with this yeast system showed that sex conferred a greater rate of adaptation to a stressful environment, but that sex had no effect on fitness in a permissive environment [15]. *S. cerevisiae*'s native *U *is very small at around 0.001 [36,46,47] and thus the genetic load imposed by any detrimental mutations is negligible; consequently this experiment was unable to evaluate the effects of sex on the clearance of detrimental mutations [36,48]. Here we have additionally elevated the mutation rate by deleting *MSH2*, a gene involved in DNA mismatch repair [36], in an attempt to increase the extent of genetic load experience by these populations.

With this yeast system comprising sexual and asexual, wild-type (WT) and mutator populations we attempt a test of the 'strict F-M' hypothesis. This states that sex functions solely to increase the efficacy with which beneficial mutations are incorporated into populations, but that sex has no bearing on the efficacy with which selection operates on detrimental mutations. We are not yet in a position to manipulate the suite of mutations that arise, and thus we instead controlled the environment of selection. We constructed two environments by manipulating osmotic and thermal stresses: a 'permissive' environment at 30°C with 0 M NaCl; and a 'stressful' environment at 37°C and 0.2 M NaCl. We suggest that that purifying selection predominantly operates in the permissive environment, and this serves to remove detrimental mutations as they arise. In the more stressful environment we suggest a greater strength of directional selection will be operating, which will serve to incorporate beneficial mutations. Of course, each environment will not be absolute in the type of selection imposed - there will likely be both types of selection in both environments. In addition, there is evidence from other yeast populations, which shows that the load of detrimental mutations will likely be enhanced in stressful environments [49,50]. We assume that directional selection is relatively stronger in the stressful environment, but that purifying selection is still important here. Under these assumptions we test the predictions for changes in fitness in environments intended to impose different types and strengths of selection pressures, with populations of varying sexual status and mutation rates, and then infer the actions of sex as related to beneficial or detrimental mutations. The first prediction arising from the strict F-M hypothesis is that under permissive conditions, where purifying selection is likely more important, sex will have no significant effect on fitness regardless of the magnitude of mutation pressure. The second prediction arising from this hypothesis is that under stressful conditions, where directional and purifying selection are important, sexual populations will display greater rates of adaptation compared with asexuals, and that an elevated mutation rate will effect the difference in rate of adaptation between sexuals and asexuals only if the supply of beneficial mutations is limiting. We evolved WT and elevated mutation rate sexual and asexual yeast populations in stressful and permissive environments in order to test the two predictions arising from the strict F-M hypothesis.

## Results

### Mutation rate manipulation effects

We first conducted assays to determine if removing *MSH2 *had any effect on fitness under these experimental conditions. The mean per mitotic generation Malthusian fitness (*m*), which is log Darwinian fitness (*w*), effect of removing *MSH2 *is negligible and non-significant at 0.0008 ± 0.0216 (0.08 ± 2.2%; ANOVA comparing sexual-WT, sexual-mutator, asexual-WT and asexual-mutator, F(2,15) = 1.42, *P *= 0.2636, *n *= 6). We then ascertained the effect of removing *MSH2 *on mutation rate, and fluctuation tests showed an increase of approximately ten-fold in the *msh2Δ *(mutator) strains compared with the WT strains at the *CAN1 *locus: WT rates were 6.91 × 10^-8 ^per base pair per generation (95% confidence limits 4.64 × 10^-8 ^to 9.50 × 10^-8^); *msh2Δ *rates were 7.32 × 10^-7 ^per base pair per generation (8.67 × 10^-7 ^to 6.06 × 10^-7^); see Additional file 1 for further details. Our estimate of a ten-fold elevation in mutation rate for *S. cerevisiae *at *CAN1 *is lower than the 35-fold value for *msh2Δ *strains reported by Zeyl and de Visser [36], and it is also lower than the 75-fold increase at the *URA3 *locus based on fluctuation tests by Grimberg & Zeyl [40]. We note our estimate is at the lower end of the spectrum reported in the literature, but it does fall within the overall *msh2Δ *mutator strength estimates of Zeyl and de Visser based on both the *CYH2 *and *URA3 *loci which were 5- and 150-fold respectively [36].

Calculating *U*, the diploid genome rate of detrimental mutation per generation, from these values is not straightforward: a per locus mutation rate does not easily transform into a per genome detrimental mutation rate [35]. We follow Drake, who also estimated *U *from fluctuation tests at the *CAN1 *locus, and use his correction factor to account for synonymous mutations [35]. The estimate of *U *for the WT strain is 0.0015, and the mutator is 0.016, which is ten-fold greater as expected given the elevated mutation rate. Our estimate of a WT *U *is intermediate to the estimates of 0.002 from Drake [35] based on the *CAN1 *locus, and an estimate of 0.0011 from Wloch *et al. *[47]. Zeyl and de Visser [36] conducted mutation accumulation tests and so were able to employ the more robust Bateman-Mukai method which gave estimates for *U *of ~0.003 and ~0.02 for WT and mutators respectively: these are comparable to our respective estimates of 0.0015 and 0.016 for these WT and mutator strains.

In sum, the deletion of *MSH2 *elevated mutation rates by ~10-fold and increased *U *by a similar magnitude (0.015) in these strains. The magnitude of increase in mutational load imposed by deleting *MSH2 *here is at the lower end of the range reported in the literature. The MD hypothesis predicts that sex only alleviates the supposed two-fold cost it imposes when *U *≥ 1 and when detrimental mutations interact with synergistic epistasis. Since *U *is only 0.016 this system is unable provide a strict test of this hypothesis. However, since mutational pressure is increased, there should be some effect on fitness. At mutation-selection balance the difference in selection coefficients among detrimental mutations cancels out and thus we may estimate load given a certain value of *U *[51]. The Haldane-Muller principal, which only applies at mutation-selection balance (as does the MD hypothesis), states this will be between *U *and 2*U *depending on the dominance/recessive nature of mutations [52]. Thus, all other things being equal, if sex serves to efficiently clear detrimental mutations, then the sexual-mutator lines are predicted to be between 1.5% to 3% greater in mean equilibrium fitness compared with the asexual-mutator lines in the permissive environment, which mostly imposes purifying selection.

### Test of the strict F-M hypothesis

#### a. **The effects of sex in the permissive environment**

**Figure 1:**
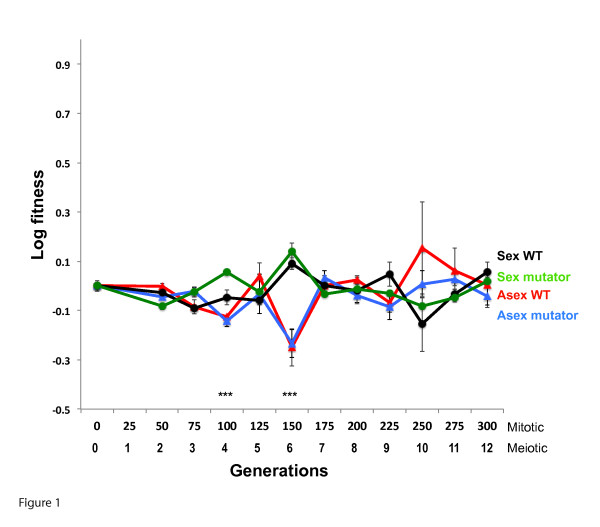
**Fitness in the permissive environment**. Malthusian (log) fitness of experimental yeast populations propagated in the permissive environment, ascertained by head-to-head competitions with the ancestor. Each point shows the mean (± SE) of triplicates for each sexual status by mutation rate treatment. The only time points where a two-way ANOVA revealed a significant effect of either sexual status or mutation rate on fitness are at generation 100 and 150 (indicated by ***) where only sexual status had a significant impact on fitness (*P *< 0.0006). There is no effect of sexual status or mutation rate on fitness at any other time point (P > 0.2). Models describing individual linear fits for each treatment did not fit the data any better than one linear fit given all the data (*P *= 0.37).

Effective population sizes were significantly different after 300 generations (ANOVA, *n *= 3, F(3,8) = 7.10, *P *= 0.0121), with the asexual-WT and asexual-mutators (mean 7.38 × 10^5 ^cells/mL) differing from the sexual-mutators (6.40 × 10^5^) as revealed by a Tukey-Kramer HSD test (α = 0.05). The sexual-WT population sizes were not different from any other treatment (α = 0.05). However, there was no difference in fitness between any treatment in the permissive environment after 300 generations (ANOVA on final fitness, *n *= 3, F(3,8) = 0.373, *P *= 0.7751; see Figure 1). Further, we found no significant difference between linear models that grouped these data by the presence or absence of sex, or mutation rate, or did not group the data at all (ANOVA, F(2,152) = 2.26, *P *= 0.1080). The slope of a model fit to all the data was not significantly different from zero (95% confidence intervals of the total fitness gain or loss span -1.3% to +9.9%; *n *= 12). These analyses show there was no significant change in fitness of any population in the permissive environment. The lack of fitness gain implies few if any beneficial mutations of large effect were fixed and thus suggests that purifying selection was likely more important here. These data show that sex had no effect on fitness under conditions where directional selection is not operating strongly.

The alternative to the strict F-M hypothesis predicts sex functions to more efficiently clear detrimental mutations - can we provide a sufficient test to falsify this idea to increase our confidence in the passive role that sex plays with regard to clearing detrimental mutations? The elevated mutation pressure of Δ*U *= 0.015 is predicted to reduce fitness by 1.5% or greater once populations are at mutation-selection equilibrium. If sex alleviates mutation pressure through more efficient mutation clearance, then the sexuals should be higher in fitness when compared to asexuals. If the difference between the sexuals and asexuals is less than 1.5% we may reject the idea that sex functions to efficiently clear detrimental mutations. The 95% confidence limit for fitness loss given the data from all populations is -1.3%, close to the -1.5% threshold predicted. If separate linear models are fit to each treatment, the difference in fitness between asexual-mutators and sexual-mutators is well below 1.5% at just 0.03% per generation, with the 95% CI of each treatment's fitness change per generation spanning -0.01% to +0.07% (asex-mutator) and -0.03% to +0.02% (sex-mutators). The difference in fitness between the sexuals and asexuals is thus ten-fold lower than predicted at equilibrium by the mutation clearance model. Together these tests suggest sex served no function in the permissive environment where weak purifying selection was mainly operating.

### b. The effects of sex in the stressful environment

**Figure 2:**
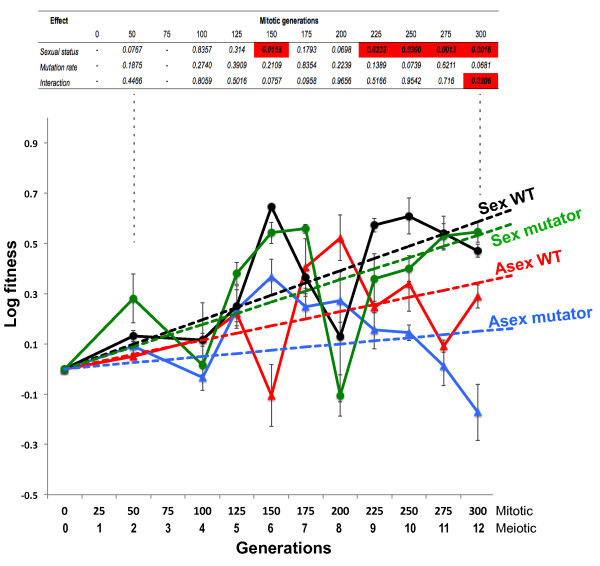
**Fitness in the stressful environment**. Malthusian (log) fitness of experimental yeast populations propagated in the stressful environment, ascertained by head-to-head competitions with the ancestor. Each point shows the mean (± SE) of triplicates for each sexual status by mutation rate treatment. The *P *values revealed by a two-way ANOVA of fitness for each time point are shown at the top of the plot, values below *P *= 0.031 are highlighted in red. Note that this analysis was conducted on each time point independently. Sexual populations have greater fitness from generation 225 onward and mutation rate does not significantly affect fitness at any time point. The best-fit linear model for each treatment is also shown. Comparisons of linear models show there is a significant effect of sexual status on fitness trajectories (*P *= 0.0013), but not mutation rate (*P *= 0.3308); there is no difference between sexual WT and sexual mutator fitness (*P *= 0.652), but a reduced probability that the observed difference in fitness trajectories between the asexual-WT and asexual-mutators was by chance (*P *= 0.0591).

In contrast to the lack of fitness change in the permissive environment, more dynamic fitness trajectories were apparent in the stressful environment (Figure 2). One of the sexual-WT populations repeatedly went extinct and was abandoned after 75 generations. It is apparent from Figure 2 that fitness fluctuates dramatically among and within treatments.

Final effective population sizes significantly differed between treatments (ANOVA, *n *= 3, F(3,8) = 10.76, *P *= 0.0052) and a Tukey-Kramer HSD test (α = 0.05) revealed that both elevated mutation treatments (mean ± se 6.71 ± 0.10 × 10^4 ^cells/mL) differed from the asexual-WT (0.40 ± 0.09 × 10^4^), and the sexual-mutator populations (7.63 ± 0.01 × 10^4^) differed from the sexual-WT (0.25 ± 0.00 × 10^4^). A two-way ANOVA examining the effects of sexual status and mutation rate, and any interaction between these, on final fitness, revealed no dramatic effect of mutation rate (F(1,10) = 13.87, *P *= 0.0681), but that sexual status had a significant effect (*P *= 0.0016). However, this analysis also revealed a significant interaction between the two main effects (*P *= 0.0206). There was no significant difference between the final fitness of the sexual-WT and sexual-mutators (*n *= 3, unequal variance *t*-test, *t*-Ratio = 1.1, *P *= 0.3576), and on average the sexual lines achieved a 59% fitness increase over the asexuals after 300 generations (mean sexuals = 0.51 ± 0.07; equivalent to a 67 ± 7% Darwinian fitness (± 95% CI); mean asexuals 0.06 ± 0.244 = 6% ± 28%). The interaction between main effects derives from differences within the asexual treatment: the asexual-mutators were significantly less fit than the asexual-WT (*n *= 3, unequal variance *t*-test, 1-tailed, *t*-Ratio = -3.1, *P *= 0.0313; *n *= 3). The mean difference between the asexual-WT and asexual-mutators was 0.46 (58%). Two of the three asexual mutator populations had similar final fitness averaging -0.04 (-4%) but the third population had a fitness of -0.44 (-64%). However, given the extent of fluctuations in fitness, an analysis of the end time-point is likely a poor estimator of general trends. Figure 2 shows the analyses of every time point by 2-way ANOVA. This reveals no other time-point with a significant interaction between sexual status and mutation rate. At no time point is there a significant effect of mutation rate on fitness. Sexual status had a more sustained effect on fitness however - after 225 generations sexual populations consistently achieved greater fitness (*P *< 0.03), regardless of mutation rate. Sequential tests of fitness at individual time points are not ideal as they are not independent, and there are also potential multiple testing issues, although here the number of tests is not large.

Modeling fitness trajectories potentially provides a better test of the strict F-M hypothesis. However, it is hard to discern an appropriate model given the erratic nature of the fitness trajectories. Overall, the fitness dynamics of the sexual and asexual treatments appears to differ; see Figure 2. Sexual populations increased in fitness over the 300 generations, with no dramatic difference in fitness trajectories between WT and mutator populations. However, the fitness increase of asexuals appears to stall at around generations150 to 200, with the asexual-mutator lines showing a seemingly earlier and greater retardation in fitness gain. The largest problem is that different treatments show different patterns of fitness change, and thus it is hard to see one model that might fit all treatments. We conducted exploratory analysis with the range of hyperbolic, exponential and power-based models described in Bolker [53], but only Ricker and Holling type III models provided any degree of fit, and these are not obviously appropriate to model these data as a whole, and thus to allow comparisons of the effect of varying treatments on fitness. We decided to model fitness trajectories with conservative simple linear functions: this in effect smoothes fluctuations and estimates mean change in fitness. It is possible to use break-point regression models, but the large spikes in fitness at certain points may lead to erroneous fits and so we did not pursue this. Comparisons of linear models show a significant effect of sexual status on fitness (likelihood ratio = 13.31572, *P *= 0.0013), but no effect of mutation rate (likelihood ratio = 2.212369, *P *= 0.3308). Within the sexual treatments there was no difference in linear trajectories between differing mutation rate treatments (likelihood ratio = 0.855309, *P *= 0.652). However, there was reduced probability that the observed difference in fitness trajectories between the asexual-WT and asexual-mutators was by chance (likelihood ratio = 5.658583, *P *= 0.0591). Figure 2 shows the best-fit linear model for each treatment.

## Discussion

This study attempted to test the somewhat unrealistic 'strict F-M' hypothesis proposing sex only functions on the efficacy with which selection operates on beneficial mutations, but that sex has no effect on detrimental mutations. We were able to directly manipulate sexual status, and the rate of mutation supply, but not the proportion of beneficial and detrimental mutations. We attempted to indirectly manipulate the proportion of beneficial and detrimental mutations by controlling the degree of environmental stress. Our conclusions are based on the assumption that both types of selection will have occurred in both environments, but that directional selection was weak in the permissive environment. It must be acknowledged that the effects of purifying and directional selection cannot be fully separated here.

### Fitness changes in the permissive environment

The lack of fitness gain in the permissive environment suggests few beneficial mutations arose and thus that directional selection was weak. The stasis in adeptness suggests purifying selection was operating however. We found no significant difference in fitness between sexual and asexual populations in the permissive environment, even under an elevated mutation rate. However, the mutation rates in the engineered mutator strains here were still not large enough for a burden to be manifest in the asexual populations. It might well be that these populations did not achieve mutation-selection balance, in which case the predictions for the fitness effects of increased mutation rate are less clear. Since we could not demonstrate that an increased fitness load was induced by the elevated mutation rate, this means we cannot effectively evaluate the function of sex with regard to mutation clearance in this permissive environment. While we cannot provide data to evaluate the MD hypothesis, the main test of interest here was the 'strict F-M' hypothesis and in line with predictions from this, sex had no effect on fitness under conditions where directional selection is not operating strongly.

### Fitness change in the stressful environment

Fitness trajectory slopes were positive for all populations evolved in the stressful environment, showing beneficial mutations were generally incorporated and that directional selection was operating. However, if purifying selection removes detrimental mutations, then this must also be operating here to some degree. In addition, it is likely that the effects of detrimental mutations were greater in the stressful than in the permissive environment, as has been shown in other experimental yeast populations [49,50]. It is thus likely that the detrimental mutation burden was larger in the stressful environment than in the permissive environment.

The higher fitness achieved by the sexual populations in the more stressful environment (regardless of mutational load) is in line with the second prediction from the strict F-M hypothesis, and supports the idea that one advantage of sexual reproduction is in the more efficient incorporation of beneficial mutations, as has been shown directly [20,24]. Since there was no difference in the extent of fitness gain between sexual-WT and sexual-mutators, we conclude that rates of beneficial mutation supply were not different in these populations, and thus by extension, that the magnitude of clonal interference was not different between the asexual-WT and asexual-mutator populations. However, there was a strong trend for difference in adaptive trajectories, and final fitness, between asexual-WT and asexual-mutators. This suggests that that selection was unable to operate as effectively in the asexual-mutator populations. One explanation for this observation is that rates of beneficial mutation incorporation differed between the two asexual treatments, but this does not tally with the fact that the treatment with the lower overall mutation rate (WT) had greater fitness. The logical explanation for this observation accounts for the effects of detrimental mutations also, and the efficacy with which they are cleared. If the effects of detrimental mutations are increased in the asexual-mutator populations, this will depress fitness gains. It seems that effective beneficial mutation incorporation and effective detrimental mutation clearance are increasingly hindered in asexuals with increasing mutation rates. In contrast, adaptation is clearly unaffected by increased mutation rates in sexual-mutator populations, which were presumably exposed to similar levels of beneficial and detrimental mutations. Thus, our data do not strongly agree with the second prediction of the strict F-M hypothesis. Rather, these observations tend to support integrative theories that describe a more sophisticated function of sex. These ideas predict that asexual populations will be retarded in adaptation because they cannot unlink beneficial mutations from detrimental ones as effectively as sexual populations can, and that this will be exacerbated with increasing mutation pressure [1,37,39]. In the stressful environment we infer that sex played a role in the both the more efficient removal of detrimental mutations and more efficient incorporation of beneficial mutations, which both will have aided adaptation. Sex may simultaneously enhance the actions of both directional and purifying selection by unlinking beneficial from detrimental mutations.

### Caveats

The variance of fitness within and among treatments in the stressful environment is large, and the various treatments show no clear evidence for fitness stabilization (though the last 75 generations appeared more consistent). It is not clear what the reason for this was, and we cannot discount that some other factor was having a large influence on fitness. That changes in effective population estimates do not correlate with changes in fitness adds to this concept. Some time points for some populations show a large drop in fitness, for example at generation 200 both sexual populations drop dramatically, only to regain fitness levels in the next experimental cycle. It is possible that some technical issue caused this, though the asexual populations were contemporary to the sexuals and did not show such a drop. These trajectories differ from those seen in similar studies using the same yeast strains in similar environments [15] where the fitness gains were more uniform. One difference from the previous study is that here populations were propagated by serial transfer and not continuous culture. Since we measured fitness by competition with the ancestor, it is feasible that undulations in fitness might be due to intransitive fitness dynamics [54]. We assayed independent lag, exponential growth and population density parameters for the ancestors and derived populations at 150 and 300 generations, and found no significant decrease in these fitness parameters, but we saw no significant increase in them either (data not shown). One difference between sexual and asexual treatments is that only the sexual lines produced haploid spores, and here recessive or semi-dominant mutations were exposed to selection. This did not occur in the asexual populations. In addition ~20% of the sexual populations were inbred, and these may have produced diploid progeny homozygous for detrimental mutations, again allowing selection to operate on these more effectively. Together these may have contributed to the more efficient removal of detrimental mutations in sexual populations, and explain these observations to some extent. Either way these are still net effects of sex. Effective population sizes in the selection experiment exceed 3 × 10^3 ^and this leads to stochastic explanations for these observations being of lesser importance. It is still possible that in smaller populations, stochastic effects such as Muller's ratchet may occur, and allow sex to more effectively clear detrimental mutations in the absence of beneficial mutations [27].

## Conclusions

Sex provided no advantage in the permissive environment, and it seems that purifying selection operated equally as efficiently in sexual and asexual populations, regardless of a ten-fold increase in mutation rate. The fact that the elevated mutation pressure induced here was not enough to produce a measurable fitness load in any population in the permissive environment should be balanced with the fact that this elevated mutation rate is comparable to the natural mutation rate of higher eukaryotes [29]. Extrapolation from these data would thus suggest that sex serves no function in higher eukaryotes residing in static environments to which they are well adapted. The data produced by these experiments, while not completely clear-cut, tend toward a rejection of the strict F-M hypothesis. These data show that sex facilitates adaptation to stressful environments, and the best explanation for the observations here is that the mechanisms of sex's advantage do not simply lie in the more efficient incorporation of beneficial mutations, but also more efficient detrimental mutation clearance. Our results agree with the recent reports from experimental inbred and outcrossed nematode populations [19]. Together, these observations fit with theory suggesting that the function of sex is potentially more sophisticated than simply operating on either beneficial or detrimental mutations [39,55]. Sex separates individual mutations, which allows selection to operate on them independently: beneficial mutations are maintained, and detrimental mutations are removed, more effectively. In these experiments sex was only of benefit in stressful environment, but was of no consequence in permissive static environment. This suggests that the extinction of asexual species is unlikely to be deterministic, but perhaps due to a failure to as effectively adapt to new environments compared with sexual species. This supports neutral models of asexual lineage longevity [56], which show that under a neutral branching framework, some long-lived asexual lineages are to be expected in the evolutionary tree of life.

The net result here is in keeping with previous work: sex speeds adaption, and these data provide another step toward understanding how sex achieves this. It is likely that most populations will experience both beneficial and detrimental mutations in most environments. It seems unrealistic to imagine that sex only functions on the efficacy with which selection operates on only one or other of these types of mutation. These data fit with an arguably more general and realistic concept that is essentially the idea originally articulated by Weismann [4], and this suggests that sex unlinks different mutations and allows selection to operate on all of them with greater efficacy, regardless of their nature.

## Methods

### Strains

The *S. cerevisiae *strains employed here were originally described by Goddard et al. [15]. In order to elevate mutation rate *MSH2 *was deleted and replaced with *URA3 *in each of the four haploids used to create the diploid mutator strains using the standard Lithium Acetate transformation method. These putative mutator diploids only differ from the wild-type mutation rate (WT) diploids at the *MSH2 *locus, and have the genotypes *ho, ura3Δ, spo11Δ, spo13Δ::kanMX4, msh2Δ::URA3 *(asexual-mutators) and *ho, ura3Δ, msh2Δ::URA3 *for (sexual-mutators).

### Mutation rates

In order to determine the effects of deleting *MSH2*, the mutation rates at the *CAN1 *locus for *MSH2 *(WT) and *msh2Δ *(mutator) haploid strains were estimated with Luria-Delbruck fluctuation tests following Lang and Murray [46]. Haploid strains were used as the majority of Canavanine resistance mutations are recessive [46]. The strains were grown at 30°C with shaking in 3 mL of SD media (0.17% yeast nitrogen base without amino acids, 2% glucose, 0.5% ammonium sulphate, 20 mg/L uracil). After 24 hours, each culture was diluted one-thousand fold, and 10 μL of the resulting mix added to 90 μL of SD media and incubated at 30°C overnight. The entirety of each sample was spread onto SD + canavanine (60 mg/L) plates and incubated at 30°C for three days. Colonies greater than 1 mm were counted as mutants under a dissecting microscope at 10 × magnification. Mutation rate at the *CAN1 *locus was calculated using the MSS-maximum likelihood method [57,58], and 95% confidence limits by the method described by Stewart [59] and Foster *et al. *[60]; see Additional file 1.

### Evolution

Each of the four ancestors was propagated in triplicate in the environments imposing purifying and directional selection to total 24 populations. The permissive environment comprised SD glucose-limiting media (0.17% yeast nitrogen base without amino acids, 0.08% glucose, 0.5% ammonium sulphate, 20 mg/L uracil) at 30°C, while the stressful environment comprised the same SD media but with an osmotic and thermal stress (1.169% NaCl and 37°C). Each population was mitotically propagated by serial batch culture of 0.04 mL into 2.96 mL four times, totaling approximately 25 generations, after which they experienced one round of sporulation (meiosis) undertaken in 0.5% Potassium Acetate, 2.5 mg/L uracil for seven days at 30°C. *S. cerevisiae *produces four haploid spores contained in an ascus, and spores from the same ascus naturally emerge in close proximity and thus have a tendency to mate to form highly inbred diploids [61]. To counter this inbreeding tendency the resulting spores were treated in order to disrupt asci and randomise the spores. Unsporulated cells were killed by re-suspending cultures in 3 mL 80 unit/mL lyticase (Sigma-Aldrich), 50 mM dithiothreitol (Sigma-Aldrich) overnight at 37°C and then adding 1% sodium dodecyl sulfate and incubating at 37°C. Asci were disrupted by then re-suspended in 3 mL β-glucuronidase solution (1100 units/mL, Sigma-Aldrich) and incubating at 37°C overnight. The washed spores were then covered with 3 mL SD media and allowed to germinate, and mate in the sexual treatment, at 30°C overnight. Asexuals were treated identically. The resulting populations were used to initiate the next round of evolution. We empirically estimated that this procedure allowed 78 ± 6% of matings to be outcrossed in the sexual lines (*n *= 6) by crossing to identical strains but that were *lys^-^, URA^+ ^*and analyzing the segregation of auxotrophic markers following Goddard *et al. *[15].

The mitosis:meiosis cycle was repeated 12 times, for a total of approximately 300 mitotic generations interspersed with 11 rounds of sporulation, which included outcrossed sex for the appropriate treatments. Effective population size was calculated to account for bottlenecks during evolution as described by Wahl and Gerrish [62].

### Fitness assays and analyses

Fitness was determined by head-to-head competitions between oppositely marked ancestral and evolved populations. Populations were grown overnight in SD media at 30°C and the resulting cultures mixed 1:1 and 0.04 mL inoculated into 2.96 mL of the appropriate competition media and temperature. Start and end ratios of the two competitors were determined by plating on YPD (1% yeast extract, 2% peptone, 2% glucose) and then replica plating onto YPD with 200 mg/L G418 (the asexuals are G418 resistant). Differences in Malthusian (log) fitness (*m*) were calculated by difference in exponential growth rates. The effects of sexual status and mutation rate, and any interaction between them, at individual time-points were analyzed by ANOVA where main effects were treated as factors. In order to model changes in fitness over the course of the experiment, fitness trajectories were analyzed with mixed effects linear models with the nlme library using the R software package, version 2.10.0 [63]. Sexual status and mutation rate treatments were modeled as fixed effects, and within-line random effects were included, which were normally distributed with a with mean of zero and standard deviation based on the variance observed. Likelihood ratio tests as implemented in the nlme package were used to compare different linear models.

## Competing interests

The authors declare that they have no competing interests.

## Authors' contributions

MG and JG designed the experiment, JG carried out the experiment, JG and MG analyzed the data and wrote the paper. Both authors read and approved the final manuscript.

## Supplementary Material

Additional file 1 **Additional methods and analyses**. This document contains further details and data concerning estimates of mutation rates by fluctuation tests. Click here for file
