# Supplementary material for: Sex enhances adaptation by unlinking beneficial from detrimental mutations in experimental yeast populations
Source: BMC Evol Biol. 2012 Mar 30;12:43. doi: 10.1186/1471-2148-12-43 (PMC3342131; doi:10.1186/1471-2148-12-43)
Supplement: Additional file 1 — Additional methods and analyses. This document contains further details and data concerning estimates of mutation rates by fluctuation tests. [file 1471-2148-12-43-S1.PDF]

Supplementary material

for

Sex enhances adaptation by unlinking beneficial from detrimental mutations in experimental yeast populations

J C Gray and M R Goddard

Fluctuation test analyses

Fluctuation tests were carried out to determine the mutation rate of the wilt-type (WT) and engineered *msh2*Δ (mutator) strains [1]. Tests were carried out based on the method provided by Lang and Murray [2]. All tests were conducted on haploids. Strains were pre-grown at 30°C with 125 rpm shaking in 3 mL of SD media (0.17% yeast nitrogen base without amino acids, 2% glucose, 0.5% ammonium sulphate, 20mg/L uracil) from a 10μL inoculum of the relevant -80°C stock. After 24 hours, each of the cultures were diluted one-thousand fold, and 10 μL of the resulting mix was added to 90 wells in a 96 well plate containing 90 μL of SD media, the remaining six wells were used for negative controls. The plate was covered and incubated with 125 rpm shaking at 30°C overnight. The population used to initiate these cultures was serially diluted to 10^{-5} and plated on YPD plates five separate times in order to determine starting numbers. As fluctuation tests require all of each culture to be plated onto selective media, but also require knowledge of total cell number in the cultures [3], it is standard practice to pool a number of cultures, plate these out on non-selective media and use these numbers as an estimate of the number of cells in those plated to detect mutants. To achieve this, 10 randomly chosen wells of each of the wild-type and mutator strains were pooled and plated at a 10^{-5} dilution on YPD plates five times, with independent dilutions. The remaining wells were plated onto SD + canavanine plates by spotting the entire 100 μL

contents onto a plate, with three spots per plate. Following Lang and Murray [2], plates were incubated at 30°C for 3 days and mutants counted under a dissecting microscope at 10× magnification. Colonies greater than 1mm in diameter were counted as mutants, and the frequency of mutants deriving from each well is tabulated in Table S1.

Number of colonies	Wt Haploid	Mut Haploid
0	0.64	0.04
1	0.17	0.10
2	0.08	0.09
3	0.05	0.01
4	0	0.01
5	0.01	0.05
6	0.01	0.05
7	0.01	0.04
8	0	0.01
9	0	0.01
10	0.01	0.04
11	0	0.05
12	0	0.04
13	0	0.03
14	0	0.01
15	0	0.01
16	0	0.03
17	0	0.03
18	0	0.03
19	0	0
20+	0.01	0.34
Total Number of Tests	78	80
Cells Per Test	6,160,000	4,920,000

Table S1. Proportion of fluctuation test cultures showing each number of mutant colonies. ‘Cells per test’ is a measure used to correct for the number of cell divisions carried out.

There are a multitude of ways of analysing data from fluctuation tests [3, 4]. Figure S1 shows a fit of the experimental data with the expectation based on mutation rates calculated by the Ma-Sandri-Sarkar Maximum Likelihood Estimator (MSS) [4]. The MMS method give a result in mutations per culture (*m*), and it is possible to simply divide this by the number of cells per test to give a mutation per cell division rate (μ) [5]. Table S2 provides a comparison of the estimate of relative mutation rates using varying methods. The MSS estimate is used in the main text as this method employs all the data and provides confidence intervals. These calculations and statistical analysis were carried out in Wolfram Mathematica 7.0 [6]. Lang and Murray [2] give a method of inferring per base pair mutation rate for canavanine resistant mutants, allowing comparison with other studies. These data are given in Table S3.

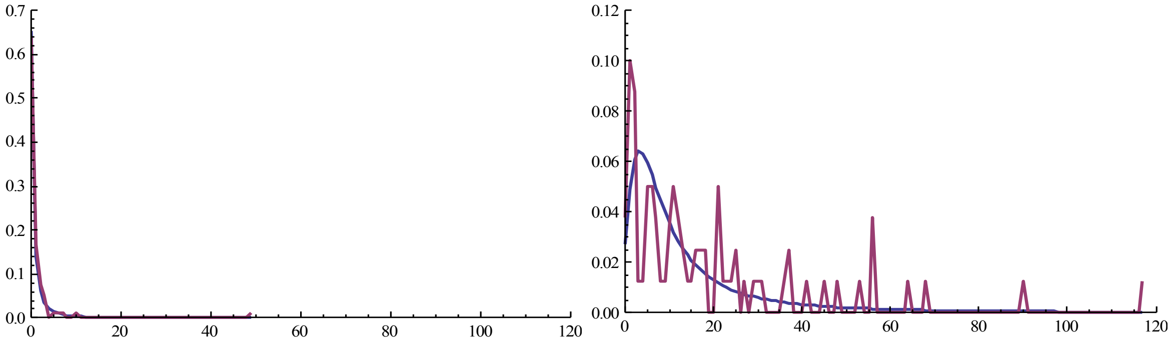

Figure S1. Experimental data and mutation rate as estimated by the MSS model, given the data. X-axis is number of mutants per culture, Y-axis is proportion of total cultures. Purple lines are experimental data; blue lines are theoretical predictions. Left panel are wild-type; right panel are mutators.

	p0	Leah Coulson	Jones Median	Koch	Drake	MSS	Luria Graph
wt haploid	7.22×10^{-8}	4.70×10^{-8}	NA	2.01×10^{-7}	1.62×10^{-8}	6.91×10^{-8}	9.53×10^{-8}
mutator haploid	6.67×10^{-7}	8.68×10^{-7}	7.82×10^{-7}	8.22×10^{-7}	1.27×10^{-7}	7.32×10^{-7}	5.49×10^{-7}
Relative mutational increase	9.24	18.48	NA	4.10	7.85	10.59	5.76

Table S2. Mutation Rate to Canavanine resistance (μ) per cell division calculated with differing methods

	Per base pair	Upper Limit	Lower Limit
wt haploid – this study	2.93×10^{-10}	4.16×10^{-10}	1.95×10^{-10}
Mutator haploid – this study	3.10×10^{-9}	3.70×10^{-9}	2.56×10^{-9}
Lang and Murray (*CAN1*)	6.44×10^{-10}	ND	ND
Lang and Murray (*URA3*)	3.80×10^{-10}	ND	ND
Zeyl *CAN1*	1.36×10^{-9}	ND	ND
Zeyl *CAN1* mutator	7.20×10^{-8}	ND	ND
Zeyl *URA3*	2.64×10^{-10}	ND	ND
Zeyl *URA3* mutator	3.92×10^{-8}	ND	ND

Table S3. Comparison of calculated mutation rates with similar studies: Lang and Murray [2] and Zeyl [7]. Support limits are based on the MSS method, and were not given by the other studies.

1. Luria SE, Delbruck M: **Mutations of bacteria from virus sensitivity to virus resistance**. *Genetics* 1943, **28**(6):491-511.
2. Lang GI, Murray AW: **Estimating the per-base-pair mutation rate in the yeast *Saccharomyces cerevisiae***. *Genetics* 2008, **178**(1):67-82.
3. Foster PL, Campbell JL, Modrich P: **Methods for Determining Spontaneous Mutation Rates**. In: *Methods in Enzymology.* vol. Volume 409: Academic Press; 2006: 195-213.
4. Rosche WA, Foster PL: **Determining mutation rates in bacterial populations**. *Methods* 2000, **20**(1):4-17.
5. Armitage P: **The statistical theory of bacterial populations subject to mutation**. *Journal of the Royal Statistical Society Series B (Methodological)* 1952, **14**(1):1-40.
6. Wolfram Research Inc.: **Mathematica**. In., 7.0 edn. Champaign, Illinois: Wolfram Research, Inc.; 2008.
7. Zeyl C, de Visser JAGM: **Estimates of the rate and distribution of fitness effects of spontaneous mutation in *Saccharomyces cerevisiae***. *Genetics* 2001, **157**(1):53-61.
